# Synthesis, characterization, and POM-protein interactions of a Fe-substituted Krebs-type Sandwich-tungstoantimonate

**DOI:** 10.1007/s00706-019-2381-5

**Published:** 2019-04-29

**Authors:** Elias Tanuhadi, Ioannis Kampatsikas, Gerald Giester, Annette Rompel

**Affiliations:** 1 Universität Wien, Fakultät für Chemie, Institut für Biophysikalische Chemie, Althanstraße 14, 1090 Wien, Austria; 2 Universität Wien, Fakultät für Geowissenschaften, Geographie und Astronomie, Institut für Mineralogie und Kristallographie, Althanstraße 14, 1090 Wien, Austria

**Keywords:** Sandwich polyoxometalates, Crystal structure, Proteins, Bioinorganic chemistry, Tungstoantimonates

## Abstract

**Abstract:**

The novel iron-substituted Krebs-type polyoxotungstate (C_12_N_4_H_11_)_4_Na_2_H_5_[(Fe(H_2_O)_3_)_2_((FeO_2_)_0.5_(WO_2_)_0.5_)_2_(β-SbW_9_O_33_)_2_] (**Fe-1**) has been synthesized using *ortho*-phenylenediamine (opda) as a precursor for the in situ formation of the counter cation 2,3-diaminophenazinium (C_12_N_4_H_11_)^+^ (2,3-DAP). **Fe-1** has been thoroughly characterized in the solid state by single-crystal X-ray diffraction (SXRD), powder X-ray diffraction (PXRD), IR spectroscopy, and elemental analysis as well as in solution by UV–Vis spectroscopy. The crystal structure of **Fe-1** reveals *π–π*-interactions between the aromatic systems of the unconventional 2,3-DAP counter cation. POM-protein interaction studies using SDS-PAGE revealed a non-proteolytic behavior of **Fe-1** towards Human Serum Albumin (HSA) as a model protein.

**Graphical abstract:**

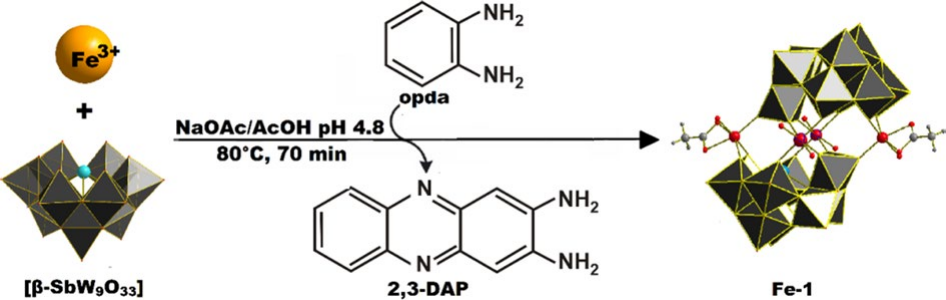

**Electronic supplementary material:**

The online version of this article (10.1007/s00706-019-2381-5) contains supplementary material, which is available to authorized users.

## Introduction

Polyoxometalates (POMs) [1] represent a broad class of anionic clusters, which are composed of metal ions in high oxidation states and linked by oxygen atoms resulting in a vast variety of unique structures. Depending on their size, charge, and composition, POM frameworks exhibit numerous different properties opening potential applications in different research fields of catalysis [2], materials science [3], and biological chemistry [4, 5] including protein crystallography [6, 7].

Among the POM family, transition metal substituted POMs (TMSPs) represent the largest group, mainly counting the subgroup of Sandwich-type POMs. Sandwich-type POMs, which are generally composed of two lacunary building blocks linked by a belt of heteroatoms, can be further divided into various subgroups, among them the Krebs-archetype.

Krebs-type POMs comprise two lone-pair containing β-Keggin lacunary fragments, e.g. [β-Sb(III)W_9_O_33_]^9−^ [8]. The first representatives of the Krebs-archetype with the general formula [M_2_(H_2_O)_6_(WO_2_)_2_(β-SbW_9_O_33_)_2_]^(14 − 2n)−^ (M = Fe^3+^, Co^2+^, Mn^2+^, Ni^2+^) were reported by Krebs and co-workers in 1997 [8], exhibiting considerable importance in the fields of both homo- and heterogeneous catalysis [9]. The use of this archetype for the synthesis of new hexagon-type Sandwich POM compounds has recently been reported [10]. As Krebs-type POMs comprise free accessible metal centers, the natural ligand-binding interactions between protein side chains and the peripheral metal centers may be of interest for POM-assisted protein crystallography [11].

Inspired by the use of opda as a precursor for the in situ generation of the unconventional 2,3-DAP counter cation, the novel iron-substituted Krebs-type Sandwich POM (C_12_N_4_H_11_)_4_Na_2_H_5_[(Fe(H_2_O)_3_)_2_((FeO_2_)_0.5_(WO_2_)_0.5_)_2_(β-SbW_9_O_33_)_2_] (**Fe-1**) has been prepared. Herein, we report on the synthesis and thorough characterization of the novel Fe-substituted Krebs-type Sandwich tungstoantimonate **Fe-1**. Regarding the scarce number of studies on the POM-protein interactions of the Krebs-POM archetype [10] and the potential use of non-proteolytic POM clusters as additives in POM-assisted protein crystallography, the POM-protein interactions of **Fe-1** with Human serum albumin (HSA) as a model protein were investigated using SDS-PAGE to assess whether **Fe-1** shows any proteolytic activity towards HSA.

## Results and discussion

### Synthesis of **Fe-1**

**Scheme 1:**
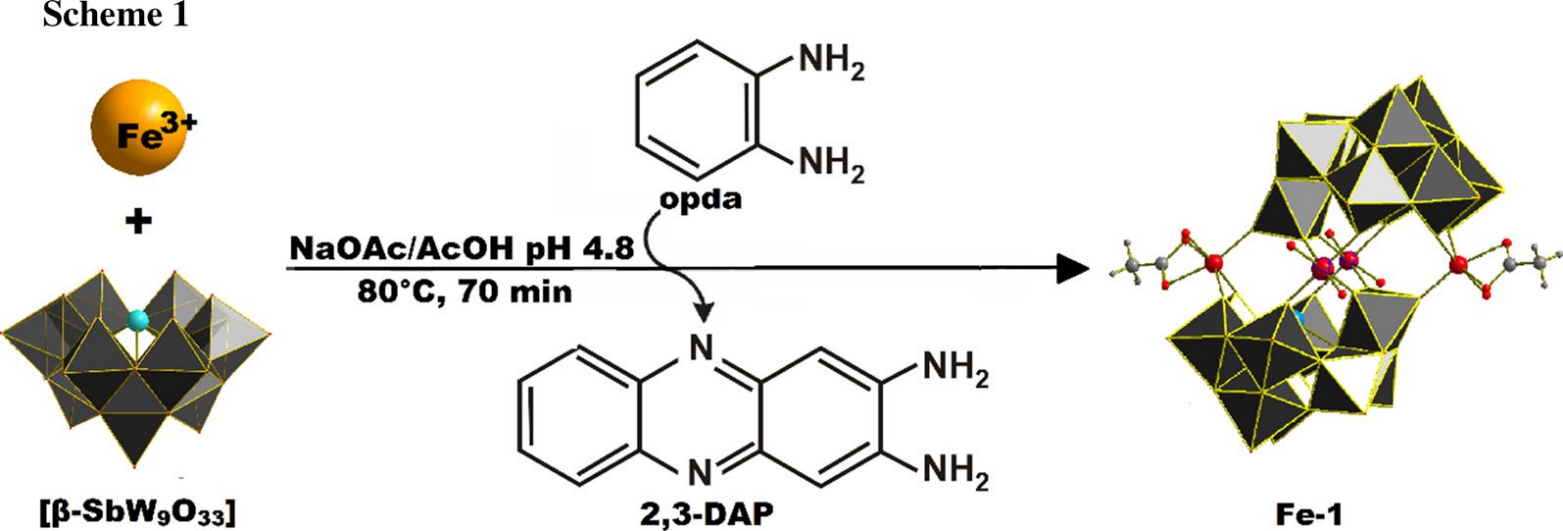
Structure and synthesis of **Fe-1**. The synthesis starts from the [β-SbW_9_O_33_] unit and FeCl_3_. Catalytic oxidation of opda by the in situ formed **Fe-1** anion leads to formation of 2,3-diaminophenazinium (2,3-DAP) which acts as a counteraction for **Fe-1**. Counter cations are omitted for clarity. Color legend: WO_6_, grey; Sb, light blue; Fe, light orange; disordered Fe/W centers, light orange with dark blue stripes; O_t_, red

An aqueous solution of Na_9_[SbW_9_O_33_] contains a mixture of [α-SbW_9_O_33_] and [β-SbW_9_O_33_] in equilibrium. It is well documented that the latter species [β-SbW_9_O_33_] dominates the equilibrium at pH values lower than 6.0 [8]. As a matter of fact, the reaction was carried out in an acetate buffer at pH 4.8. Upon addition of opda to a warm aqueous acidic reaction mixture of Na_9_[SbW_9_O_33_] and FeCl_3_, the initially yellow solution gradually turned dark red indicating the oxidation of opda to 2,3-diaminophenazine (2,3-DAP) catalyzed by the in situ formed **Fe-1** Krebs-POM. Cooling of the reaction mixture to room temperature resulted in the formation of dark red crystal plates consisting of polyanion **Fe-1** (Scheme 1).

### Crystal structure of **Fe-1**

**Fig. 1:**
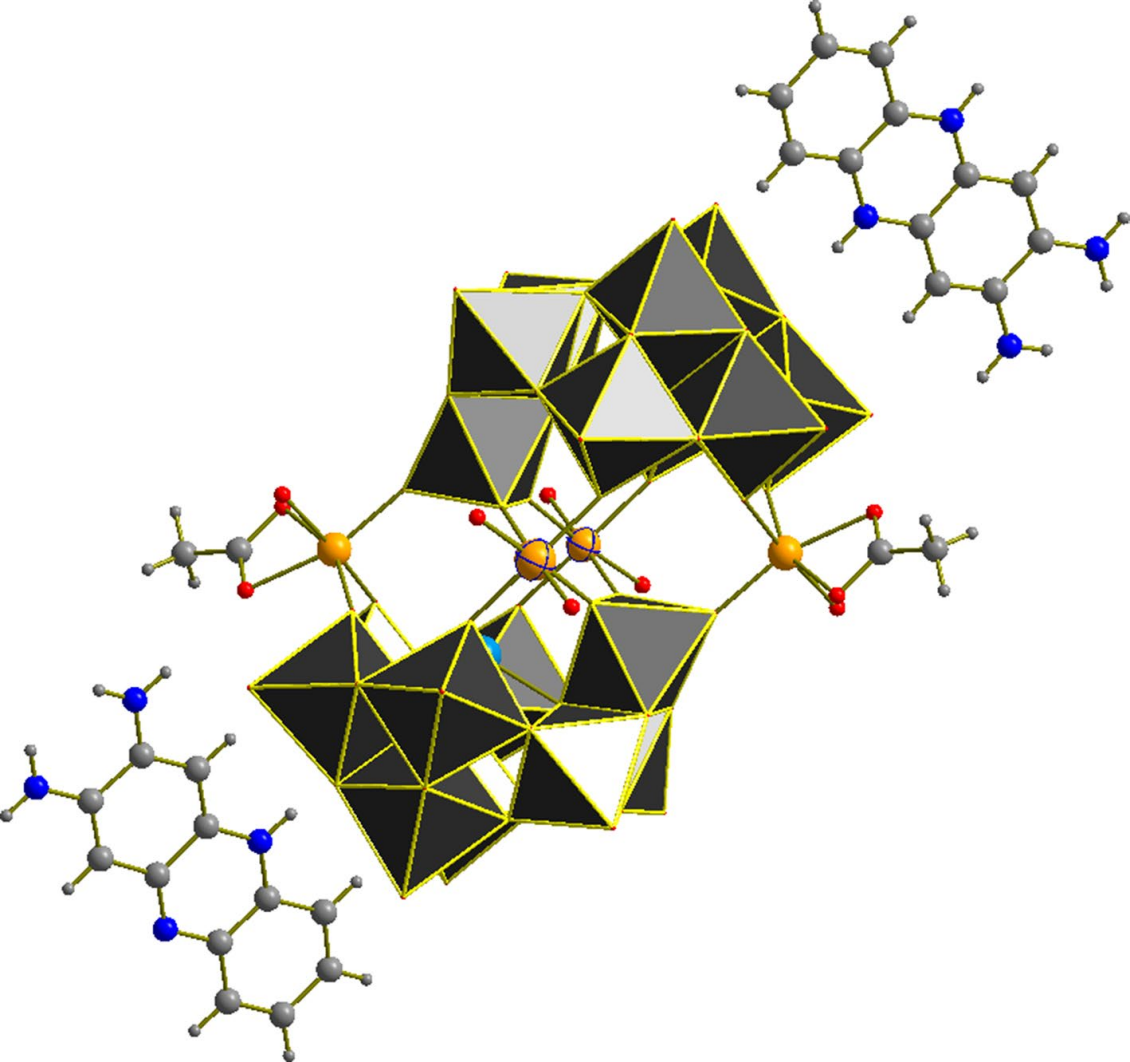
Polyhedral representation of **Fe-1**. WO_6_, grey; Sb, light blue; Fe, light orange; disordered Fe/W centers, light orange with dark blue stripes; O_t_, red; C, light grey; N, blue

Single crystal X-ray diffraction (SXRD) studies were performed on **Fe-1** revealing a Krebs-type structure which crystallizes in the triclinic space group **P-1**. The crystal structure of **Fe-1** exhibits two [β-SbW_9_O_33_] lacunary species linked by two Fe(III) metal centers at the peripheral sites and two W(VI) centers which show a 50:50 disorder with Fe(III) at the inner position of the linking belt. Regarding the synthetic conditions of **Fe-1**, which include the use of an acidic buffer (pH = 4.8), the disorder with tungsten is in accordance with the results for the disordered alpha-arsenotungstate compounds observed at lower pH values, reported by Kortz et al. in 2001 [12] as well as the disordered Krebs-type tungstoantimonates recently reported by our group [10]. The peripheral iron centers exhibit a distorted octahedral coordination environment with one acetate ligand and one H_2_O molecule coordinated to the metal center and Fe–O bond lengths ranging from 1.9271(1) at the inner site of the belt to 2.139(1) Å between the peripheral iron centers and the H_2_O ligand at the peripheral belt positions (Fig. 1).

**Fig. 2:**
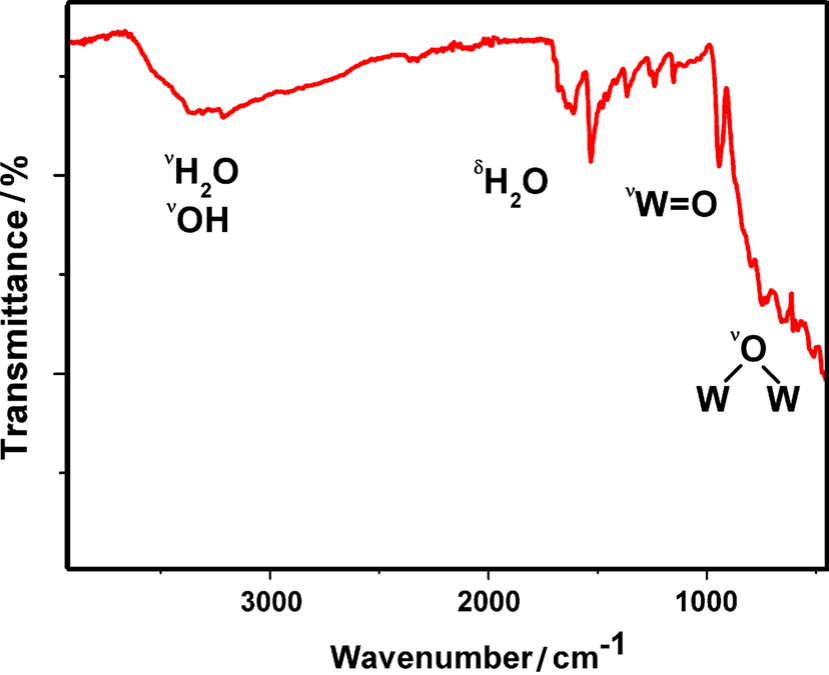
IR spectrum of (C_12_N_4_H_11_)_4_Na_2_H_5_[(Fe(H_2_O)_3_)_2_((FeO_2_)_0.5_(WO_2_)_0.5_)_2_(β-SbW_9_O_33_)_2_] (**Fe-1**)

Besides SXRD, **Fe-1** was also characterized in the solid state by powder X-ray diffraction (PXRD) (Fig. S1), ATR-IR spectroscopy (Fig. 2), and elemental analysis.

### UV–Vis spectrum of **Fe-1**

**Fig. 3:**
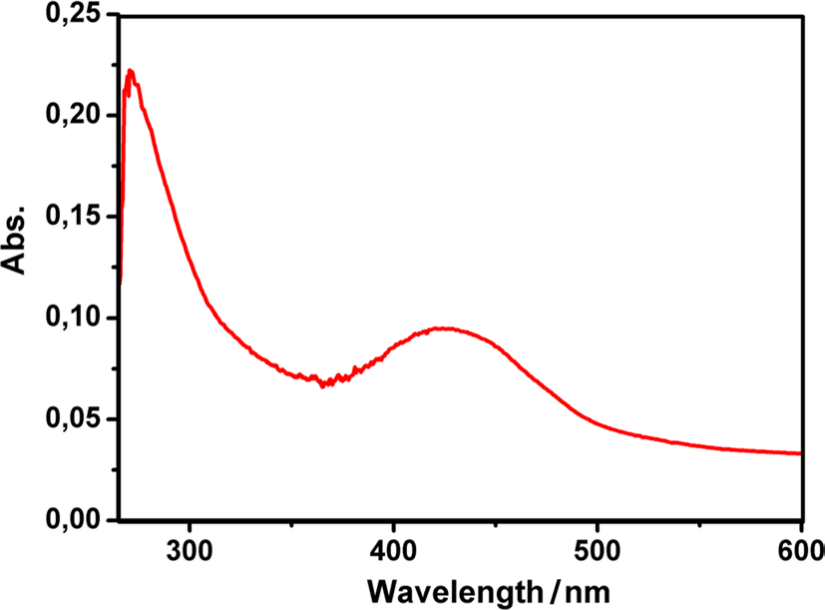
UV–Vis-spectrum of **Fe-1** (5 × 10^−6^ M) in 10 mM NaOAc buffer pH 5.5 showing typical O → W ligand—to—metal charge-transfer (271 nm) and aromatic transitions (423 nm)

The UV–Vis spectrum of **Fe-1** exhibits two major peaks, one at 271 nm corresponding to the pπ(O_b_) → dπ*(W) ligand-to-metal charge-transfer transition typical for the Keggin-type framework [13], whereas a second absorption maximum at 423 nm can be attributed to the aromatic transitions of the 2,3-DAP counter cations present in the structure [14] (Fig. 3).

### POM-protein interactions

**Fig. 4:**
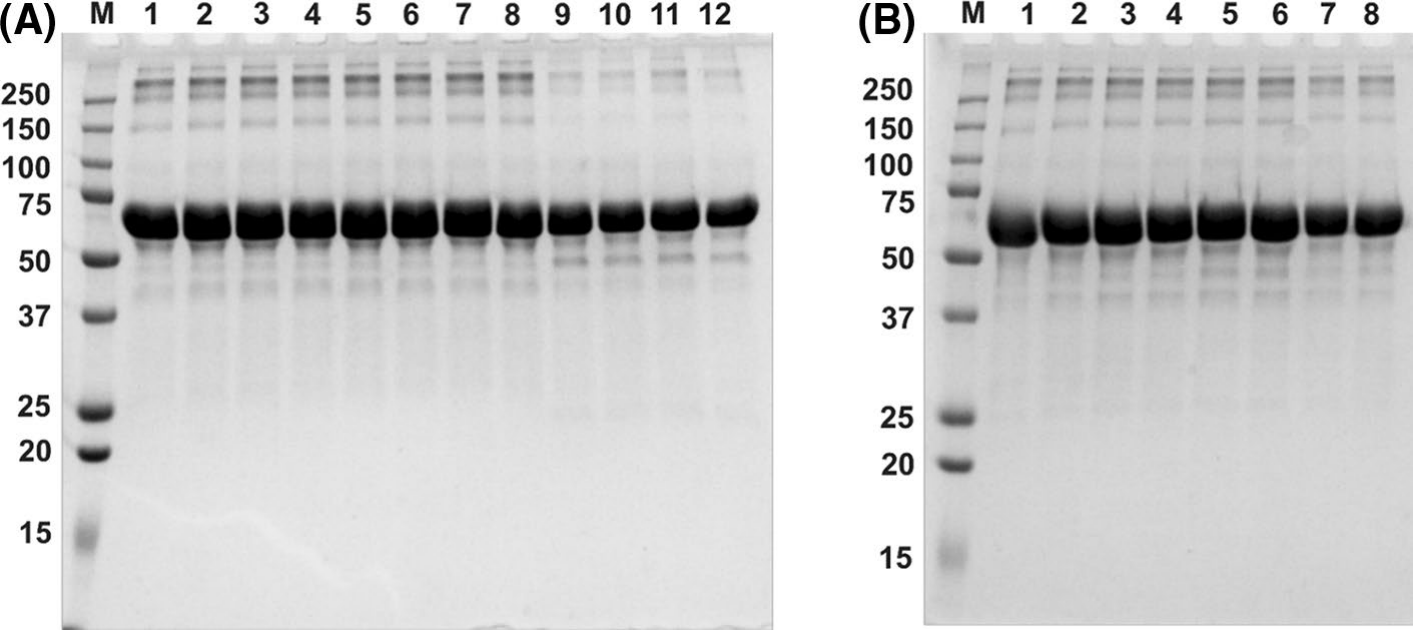
HSA incubated with **Fe-1** for 30 min at 20 °C (1–4), at 37 °C (5–8), and at 65 °C (9–12). **1)** 5 µg HSA without **Fe-1**, **2)** 1:1 HSA:POM, **3)** 1:10 HSA:POM, **4)** 1:100 HSA:POM., **5)** 5 µg HSA without **Fe-1**, **6)** 1:1 HSA:POM, **7)** 1:10 HSA:POM, **8)** 1:100 HSA:POM, **9)** 5 µg HSA without **Fe-1**, **10)** 1:1 HSA:POM, **11)** 1:10 HSA:POM, **12)** 1:100 HSA:POM in NaOAc buffer [10 mM] 5.5 pH **A)** HSA with **Fe-1** after 30 min **B)** HSA with **Fe-1** after 3 days

Considering the known catalytic activity of Fe(III) as a Lewis acid, the POM-protein interactions of the peripheral Fe(III) metal centers of **Fe-1** with human serum albumin (HSA) as a model protein were investigated to assess whether **Fe-1** exhibits any proteolytic activity. SDS-PAGE was performed on reaction mixtures of HSA and **Fe-1** in a NaOAc buffer [10 mM] pH 5.5 to ensure a stable more accessible acidic conformation of the model protein [15]. The results revealed no hydrolytic activity of **Fe-1** towards the peptide bonds of the model protein even at 65 °C and 100-fold excess of the POM compound indicated by intact protein bands at 66 kDa (Fig. 4). This is in good accordance with our previous results reported for the isostructural manganese- and zinc-substituted DAP–POM derivatives [10].

## Conclusion

In conclusion, the synthetic pathway presented in this work may open new perspectives for the preparation of novel Krebs-POM archetypes exhibiting unconventional counter cations. The interactions of the Krebs-POM compound **Fe-1** with HSA as a model protein have been investigated and the non-proteolytic behavior of **Fe-1** may be interesting for further POM-protein interaction studies ultimately perhaps opening novel perspectives in the field of POM-assisted protein crystallography.

## Experimental

All reagents were obtained commercially from Aldrich, of high-purity grade and were used as purchased without further purification. Na_9_[*B*-α-SbW_9_O_33_] was prepared according to the literature procedure reported by Bösing et al. [8]. X-ray intensity data were measured on a Bruker  X8 APEX2 diffractometer equipped with a multilayer monochromator, Mo K/α INCOATEC micro focus sealed tube and Oxford cooling device. The following software was used: Bruker SAINT software package [16] using a narrow-frame algorithm for frame integration, OLEX2 [17] for structure solution, refinement, molecular diagrams and graphical user-interface, Shelxle [18] for refinement and graphical user-interface SHELXS-2013 [19] for structure solution, SHELXL-2013 [20] for refinement. Experimental data and the CCDC-Code are provided in Table S1. Crystal data, data collection parameters, and structure refinement details are given in Tables S2 and S3 of the electronic supporting information. X-ray powder diffraction measurements were performed on a Bruker D8 ADVANCE diffractometer, Cu Kα radiation, *λ* = 1.54,056 Å, LYNXEYE silicon strip detector and SolX energy dispersive detector, variable slit aperture with 12 mm, 5° ≤ 2*θ* ≤ 40°. Attenuated total reflection Fourier-transform Infrared Spectroscopy: all spectra were recorded on a Bruker Tensor 27 IR Spectrometer equipped with a single-reflection diamond-ATR unit. Frequencies are given in cm^−1^, intensities denoted as w = weak, m = medium, s = strong. Elemental analysis (C, H, N, O) was performed at Mikroanalytisches Laboratorium, Fakultät für Chemie, Universität Wien using the 2400 CHN Elemental Analyzer and the EA 3000, respectively. UV–Vis spectra were collected on a Shimadzu UV 1800 spectrophotometer. The spectra were recorded in 10 mM NaOAc buffer pH 5.5. SDS-PAGE was performed according to a standard procedure [21] using Precision Plus Protein Standard Dual Color (Bio-Rad) as molecular weight marker. Samples were applied to 14% polyacrylamide gels under reducing conditions. The sample amount loaded onto the gel was 5 μg. Gels were stained with Coomassie Brilliant Blue. Imaging of the gels was applied with Gel Doc™ XR of BIO-RAD. Human serum albumin (HSA) (5 µg) was mixed with 1, 10, and 100 equivalents of **Fe-1** in 10 mM NaOAc buffer pH 5.5 and incubated at three different temperatures (20, 37, and 65 ºC).

### (C_12_N_4_H_11_)_4_Na_2_H_5_[(Fe(H_2_O)_3_)_2_((FeO_2_)_0.5_(WO_2_)_0.5_)_2_(β-SbW_9_O_33_)_2_] (***Fe-1***)

To a stirred solution of 215 mg Na_9_[*B*-α-SbW_9_O_33_] (0.05 mmol) in 20 cm^3^ aqueous sodium acetate buffer (0.5 M NaOAc/AcOH, pH 4.8), 81 mg FeCl_3_**·**6 H_2_O (0.3 mmol) was added. The resulting orange reaction mixture was stirred at 70 °C for 10 min. o*rtho*-Phenylenediamine (opda, 21 mg, 0.2 mmol) was added to the reaction solution and the mixture was stirred for further 60 min at 85 °C. A color change from orange to dark red over the time period of 60 min was noticed. Dark red crystal plates of **Fe-1** were obtained upon cooling the filtered reaction mixture to room temperature and further evaporation at 18 °C gave a total yield of 60% based on W after 3 days. IR (ATR): $\bar{v}$  = 3363.4 (w), 3260.6 (w), 1635.1 (m), 1509.2 (m), 1400.4 (m), 1233.4 (s), 1152.1 (s), 935.8 (s), 744.5 (s) cm^−1^.

## Electronic supplementary material

Below is the link to the electronic supplementary material.

Supplementary material 1 (DOCX 80 kb)

Supplementary material 2 (PDF 232 kb)
