# Supplementary material for: Synthesis, characterization, and POM-protein interactions of a Fe-substituted Krebs-type Sandwich-tungstoantimonate
Source: Monatsh Chem. 2019 Apr 29;150(5):871–5. doi: 10.1007/s00706-019-2381-5 (PMC6533222; doi:10.1007/s00706-019-2381-5)
Supplement: Supplementary file 1 — Supplementary material 1 (DOCX 80 kb) [file 706_2019_2381_MOESM1_ESM.docx]

**ELECTRONIC SUPPLEMENTARY INFORMATION**

Synthesis, characterization and POM-protein interactions of a Fe-substituted Krebs-type Sandwich-polyoxotungstate

**Elias Tanuhadi^1^ ● Ioannis Kampatsikas^1^ ● Gerald Giester^2^ ● Annette Rompel^1,^ ***

^*^correspondence to annette.rompel@univie.ac.at

^1^Universität Wien, Fakultät für Chemie, Institut für Biophysikalische Chemie, Althanstraße 14, 1090 Wien, Austria. www.bpc.univie.ac.at

^2^ Universität Wien, Fakultät für Geowissenschaften, Geographie und Astronomie, Institut für Mineralogie und Kristallographie, Althanstraße 14, 1090 Wien, Austria

**Content**

[**1.** **Single-Crystal X-ray Diffraction** 3](#_Toc532914466)

[**2.** **Powder X-ray Diffraction** 4](#_Toc532914467)

# **Single-Crystal X-ray Diffraction**

**Table S1:** Experimental parameter and CCDC-Code.

| **Sample** | **Machine** | **Source** | **T** | **Detector Distance** | **Time/ Frame** | **#Frames** | **Frame width** | **CCDC** |
| --- | --- | --- | --- | --- | --- | --- | --- | --- |
|  |  |  | [K] | [mm] | [s] |  | [°] |  |
| **Fe-1** | Bruker X8 | Mo | 200 | 35 | 60 | 795 | 1.5 | 1885860 |

**Table S2:** Sample and crystal data **(C_12_N_4_H_11_)_4_Na_2_H_5_[(Fe(H_2_O)_3_)_2_((FeO_2_)_0.5_(WO_2_)_0.5_)_2_(β-SbW_9_O_33_)_2_]** **Fe-1.**

| **Chemical formula** | C_52_H_68_Fe_3_N_16_Na_2_O_96_Sb_2_W_19_ | **Crystal system** | triclinic | |
| --- | --- | --- | --- | --- |
| **Formula weight [g/mol]** | 6403,4 | **Space group** | *P-1* | |
| **Temperature [K]** | 200 | **Z** | 1 | |
| **Measurement method** | \w scans | **Volume [Å^3^]** | 3339.3(3) | |
| **Radiation (Wavelength [Å])** | MoKα (λ = 0.71073) | **Unit cell dimensions [Å] and [°]** | 12.6079(7) | 95.124(4) |
| **Crystal size / [mm^3^]** | 0.06 × 0.04 × 0.02 |  | 13.9504(8) | 93.645(3) |
| **Crystal habit** | clear dark red block |  | 20.4458(12) | 110.423(3) |
| **Density (calculated) / [g/cm^3^]** | 3184 | **Absorption coefficient / [mm^-1^]** | 17,112 | |
| **Abs. correction Tmin** | 0,5358 | **Abs. correction Tmax** | 0,7465 | |
| **Abs. correction type** | multi-scan | **F(000) [e^-^]** | 2868 | |

**Table S3:** Data collection and structure refinement of **(C_12_N_4_H_11_)_4_Na_2_H_5_[(Fe(H_2_O)_3_)_2_((FeO_2_)_0.5_(WO_2_)_0.5_)_2_(β-SbW_9_O_33_)_2_]** **Fe-1.**

| **Index ranges** | -15 ≤ h ≤ 15, -16 ≤ k ≤ 16, -24 ≤ l ≤ 24 | **2-Theta range for data collection [°]** | 3.924 to 50.698 | |
| --- | --- | --- | --- | --- |
| **Reflections number** | 67242 | **Data / restraints / parameters** | 12183/39/869 | |
| **Refinement method** | Least-squares | **Final R indices** | all data | R1 = 0.0549, wR2 = 0.0952 |
| **Function minimized** | Σ w(F_o_^2^ - F_c_^2^)^2^ |  | I>2σ(I) | R1 = 0.0379, wR2 = 0.0891 |
| **Goodness-of-fit on F^2^** | 1,033 | **Weighting scheme** | w=1/[σ^2^(F_o_^2^)+(0.0466P)^2^+0.5788P] | |
| **Largest diff. peak and hole [e Å^-3^]** | 1.87/-4.41 |  | where P=(F_o_^2^+2F_c_^2^)/3 | |

# **Powder X-ray Diffraction**


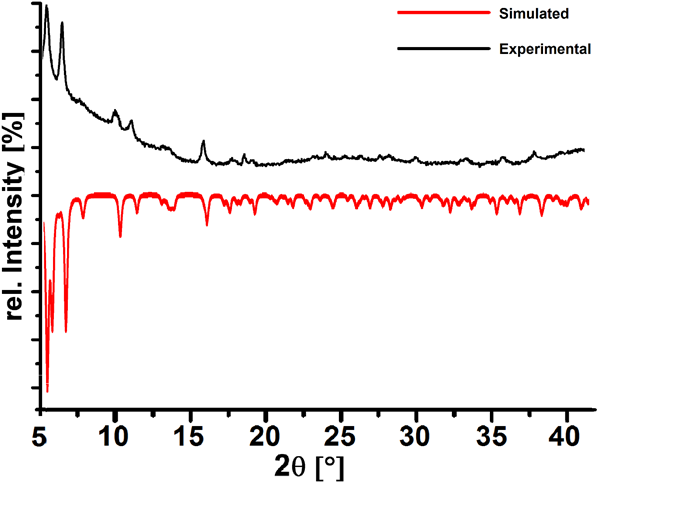


**Figure S1:** Comparison of the experimental and simulated PXRD patterns of **Fe-1**.
