# Supplementary material for: Synthesis, characterization, and POM-protein interactions of a Fe-substituted Krebs-type Sandwich-tungstoantimonate
Source: Monatsh Chem. 2019 Apr 29;150(5):871–5. doi: 10.1007/s00706-019-2381-5 (PMC6533222; doi:10.1007/s00706-019-2381-5)
Supplement: Supplementary file 2 — Supplementary material 2 (PDF 232 kb) [file 706_2019_2381_MOESM2_ESM.pdf]

ELECTRONIC SUPPLEMENTARY INFORMATION

Synthesis, characterization and POM-protein interactions of a Fe-substituted Krebs-type Sandwich-polyoxotungstate

Elias Tanuhadi[1] ● Ioannis Kampatsikas[1] ● Gerald Giester[2] ● Annette Rompel[1, *]

[*]correspondence to annette.rompel@univie.ac.at

[1]Universität Wien, Fakultät für Chemie, Institut für Biophysikalische Chemie, Althanstraße 14, 1090 Wien, Austria. www.bpc.univie.ac.at

[2] Universität Wien, Fakultät für Geowissenschaften, Geographie und Astronomie, Institut für Mineralogie und Kristallographie, Althanstraße 14, 1090 Wien, Austria

Content

1. **Single-Crystal X-ray Diffraction** 3
2. **Powder X-ray Diffraction** 4

1. Single-Crystal X-ray Diffraction

Table S1: Experimental parameter and CCDC-Code.

Sample	Machine	Source	T	Detector Distance	Time/ Frame	#Frames	Frame width	CCDC
			[K]	[mm]	[s]		[°]	
**Fe-1**	Bruker X8	Mo	200	35	60	795	1.5	1885860

Table S2: Sample and crystal data **$(C_{12}N_4H_{11})_4Na_2H_5[(Fe(H_2O)_3)_2((FeO_2)_{0.5}(WO_2)_{0.5})_2(\beta\text{-}SbW_9O_{33})_2]$ Fe-1.**

**Chemical formula**	$C_{52}H_{68}Fe_3N_{16}Na_2O_{96}Sb_2W_{19}$	**Crystal system**	triclinic	
**Formula weight [g/mol]**	6403,4	**Space group**	*P-1*	
**Temperature [K]**	200	**Z**	1	
**Measurement method**	\w scans	**Volume [Å^3]**	3339.3(3)	
**Radiation (Wavelength [Å])**	MoKα (λ = 0.71073)	**Unit cell dimensions [Å] and [°]**	12.6079(7)	95.124(4)
**Crystal size / [mm^3]**	0.06 × 0.04 × 0.02		13.9504(8)	93.645(3)
**Crystal habit**	clear dark red block		20.4458(12)	110.423(3)
**Density (calculated) / [g/cm^3]**	3184	**Absorption coefficient / [mm^{-1}]**	17,112	
**Abs. correction Tmin**	0,5358	**Abs. correction Tmax**	0,7465	
**Abs. correction type**	multi-scan	**F(000) [e$^-$]**	2868	

Table S3: Data collection and structure refinement of **$(C_{12}N_4H_{11})_4Na_2H_5[(Fe(H_2O)_3)_2((FeO_2)_{0.5}(WO_2)_{0.5})_2(\beta\text{-}SbW_9O_{33})_2]$ Fe-1.**

**Index ranges**	$-15 \le h \le 15$, $-16 \le k \le 16$, $-24 \le l \le 24$	**2-Theta range for data collection [°]**	3.924 to 50.698	
**Reflections number**	67242	**Data / restraints / parameters**	12183/39/869	
**Refinement method**	Least-squares	**Final R indices**	all data	R1 = 0.0549, wR2 = 0.0952
**Function minimized**	$\Sigma\ w(F_o^2 - F_c^2)^2$		I>2σ(I)	R1 = 0.0379, wR2 = 0.0891
**Goodness-of-fit on F^2**	1,033	**Weighting scheme**	$w=1/[\sigma^2(F_o^2)+(0.0466P)^2+0.5788P]$	

**Largest diff. peak and hole [e Å^{-3}]**	1.87/-4.41		where $P=(F_o^2+2F_c^2)/3$

2. Powder X-ray Diffraction

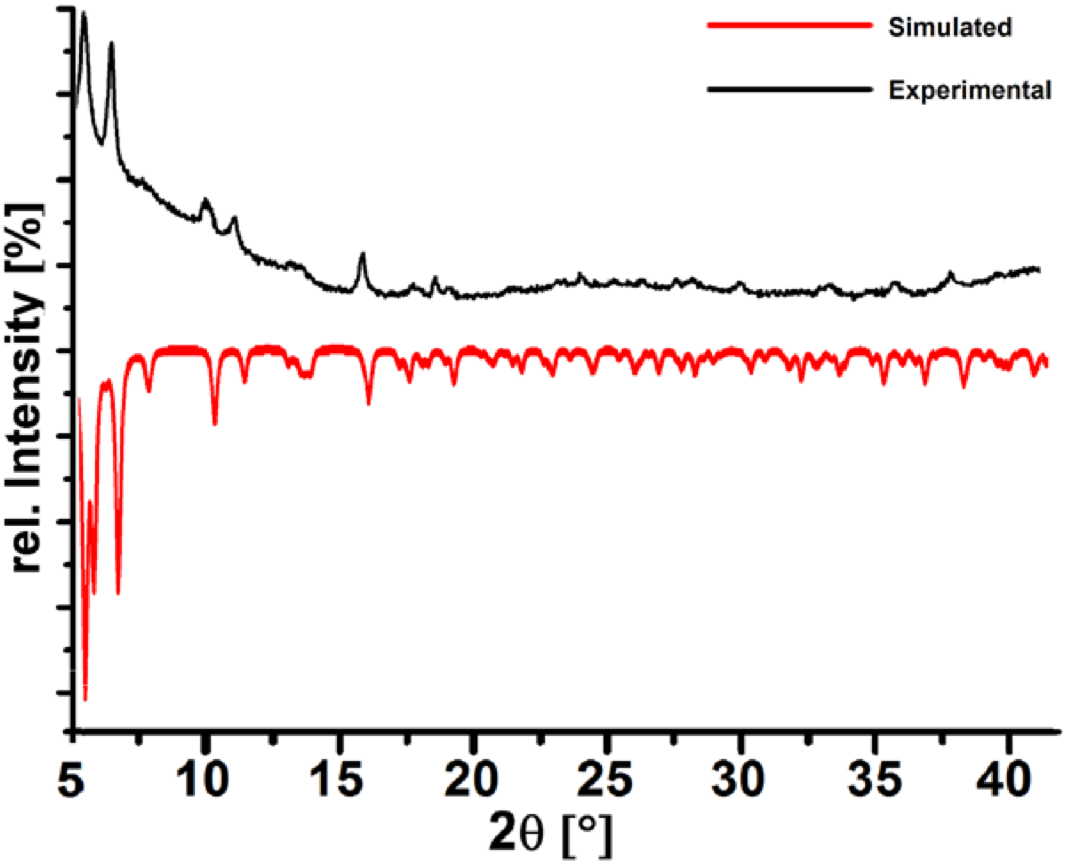

Figure S1: Comparison of the experimental and simulated PXRD patterns of **Fe-1**.
